# Cultural Adaptation of Cognitive Stimulation Therapy for Māori with Dementia (CST-Māori)

**DOI:** 10.1007/s10823-025-09527-y

**Published:** 2025-03-24

**Authors:** Makarena Dudley, Kathy Peri, Tai Kake, Gary Cheung

**Affiliations:** 1 School of Psychology, Faculty of Science, The University of Auckland, Auckland, New Zealand; 2 School of Nursing, Faculty of Medical and Health Sciences, The University of Auckland, Auckland, 1010 New Zealand; 3 Department of Psychological Medicine, School of Medicine, Faculty of Medical and Health Sciences, The University of Auckland, Auckland, New Zealand

**Keywords:** Cognitive Stimulation Therapy, Māori, Dementia, Indigenous

## Abstract

Māori are the indigenous people of Aotearoa New Zealand. Cognitive Stimulation Therapy (CST) was initially developed in the UK, lacking in Māori cultural content and values. Cultural adaptation is needed to ensure Māori with dementia can benefit from this evidence-based treatment. This paper reports the outcome of a project aimed to adapt CST for Māori. We followed the five phases of international guidelines using the formative method for adapting CST to other cultures, including a critical cultural examination of the 18 CST principles. We piloted two CST-Māori programmes and collected pre- and post-outcome measures using the RUDAS and the World Health Organization Quality of Life (WHOQOL-BREF) questionnaire. Written qualitative feedback was sought from participants and their family at the end of the CST-Māori programme. A total of 15 Māori (female: 53.3%) participated in the two programmes. Their mean age was 75.9 years (SD = 6.6) and mean baseline RUDAS scores was 17.7 (SD = 2.3). There was a statistically significant improvement in cognition (RUDAS: pre = 17.7, post = 19.4, *p* = 0.003) and in the WHOQOL subscales of physical (pre = 75.9, post = 88.5, *p* = 0.003), psychological (pre = 72.7, post = 81.3, *p* = 0.024) and environment (pre-80.6, post = 88.0, *p* = 0.006). Written feedback confirmed the acceptability of this culturally adapted programme by Māori living with dementia and their whanau (families). CST was successfully adapted for Māori with dementia. It is a culturally acceptable cognitive intervention and preliminary data confirmed the effectiveness of CST-Māori in improving cognition and quality of life.

## Introduction

Aotearoa New Zealand is a bicultural country with Māori, its Indigenous people, making up 17.8% of its population. Its remaining population is comprised of 67.8% European, 17.3% Asian, and 8.9% Pacific Peoples (Statistics New Zealand, 2024). Between 2017 and 2019 the average life expectancy for Māori males was 73.4 years and 77.1 years for Māori females, an increase of 3.1 years and 2.0 years respectively compared to the period between 2005 and 2007 (Statistics New Zealand, 2021). This increase in the number of Māori living longer means a likely increase in age-related issues such as cognitive decline and dementia (referred to as *mate wareware* by Māori). There is some evidence suggesting that dementia prevalence is higher in Māori than New Zealand European (Cheung et al., 2022; Ryan et al., 2022) and that Māori with dementia present 5–8 years earlier than New Zealand European (Cullum et al., 2018). A previous study found Māori have a lower mortality risk than New Zealand Europeans following their diagnosis of dementia in a memory service (Cullum et al., 2020). This finding could have significant implications in that Māori may live longer with dementia, which could put additional stress on *whānau* (family) supporting an elder with dementia. The ongoing systemic issue of inequities in health between Māori and non-Māori will add to this burden (Dyall, 2014).

Cognitive Stimulation Therapy (CST) is an evidence based non-pharmacological treatment for people living with mild to moderate dementia (Woods et al., 2012, 2023). It is a structured and manualized group cognitive intervention that involves 14 sessions of themed activities (Table 1). Each session runs for 50–60 min, twice a week for seven weeks. CST is informed by 18 principles (Table 2) which aim to actively stimulate and engage people living with dementia, whilst providing a social environment of six to eight participants led by two facilitators. CST has been shown to improve cognition, quality of life and communication; and is recommended for people living with mild to moderate dementia in the UK’s NICE dementia guidelines (National Institute for Health and Clinical Excellence, 2018). The evidence for CST on cognition is also highlighted in the Lancet commissioned report on dementia prevention, intervention, and care (Livingston et al., 2017).

**Table 1:** The 15 activity sessions in the Māori CST programme

UK CST Programme		CST- Māori Programme	
**Session 1**	Physical games	**Session 1** Wāhanga tuatahi	Whakawhanaungatanga-sharing and building family and tribal connections
**Session 2**	Sound	**Session 2** Wāhanga tuarua	Orientation
**Session 3**	Childhood	**Session 3** Wāhanga tuatoru	Childhood
**Session 4**	Food	**Session 4** Wāhanga tuawha	Physical games
**Session 5**	Current affairs	**Session 5** Wāhanga tuarima	Sounds
**Session 6**	Faces/scenes	**Session 6** Wāhanga tuaono	Kai (food)
**Session 7**	Associated words	**Session 7** Wāhanga tuawhitu	Current affairs
**Session 8**	Being creative	**Session 8** Wāhanga tuawaru	Faces/scenes
**Session 9**	Categorising objects	**Session 9** Wāhanga tuaiwa	Word association
**Session 10**	Orientation	**Session 10** Wāhanga tekau	Being creative
**Session 11**	Using money	**Session 11** Wāhanga tekau mā tahi	Categorising objects
**Session 12**	Number games	**Session 12** Wāhanga tekau mā rua	Using money
**Session 13**	Word games	**Session 13** Wāhanga tekau mā toru	Number Games
**Session 14**	Team games	**Session 14** Wāhanga tekau mā whā	Word games
		**Session 15** Wāhanga tekau mā rima	Team quiz and graduation

**Table 2:** The 18 principles and suggested Māori terms in the cognitive stimulation Therapy-Māori (CST-Māori) programme

CST Principles	CST-Māori Principles	Explanations
1. Person-centred	Mana Tangata	Seeing the person and their uniqueness
2. Respect	Whakaute	Respect and dignity for all
3. Building/strengethening relationships	Whakatupu/Whakakahatia Whakawhanaungatanga	Becoming friends Meaningful
4. Maximising potential	Whakakahatia Te Āheitanga Tino Rangatiratanga	Optimise the learning environment to support people’s potential
5. Mental stimulation	Whakamanawa Te Hihiritanga	Improving cognition and communication through mentally stimulating discussion
6. New ideas, thoughts and associations	Whakaaro Hōu Me Ngā Tōpūtanga Ngā Whakaaro Hōu	Encouraging new ideas and opinions by making new semantic connections (i.e., making new connections between ideas or words)
7. Using orientation sensitively and implicitly	Ahunga	Integrating orientation information into general discussion
8. Opinions rather than facts	Whakaaro	
9. Using reminiscence as a link to the here and now	Te Maumahara Hei Hono Ki Nāianei Hokinga Mahara	Comparing old and new to promote orientation
10. Providing triggers to aid recall	Taputapu Ārahi Maumahara Maumahara	Supporting learning through multisensory cues and an information board
11. Stimulating language	Reo Whakamanawa Kōrero Tahi	Promoting communication and conversation
12. Stimulating executive functioning	Whakamanawa	Using activities to support planning and organising thoughts
13. Implicit rather than explicit learning	Nga Huarahi Akoranga Takaāwhio	Let learning and remembering happen naturally
14. Continuity and consistency between sessions	Nga Whakaritenga	Using consistency of sessions to help continuity and familiarity
15. Involvement and inclusion	Mahi Tahi Me Whakawhanaungatanga	Keep everyone involved
16. Choice	Kowhiri	Activities are flexible and should be adapted for the participants
17. Fun	Whakangahau	Make it fun and enjoyable
18. Physical movement	Tinana Kori	Exercising motor skills through movements and games

CST was initially developed in the UK and therefore lacking in Māori cultural content, values, and practices. Cultural adaptation is needed to ensure Māori living with dementia will access, engage with, and benefit from this evidence based treatment. Internationally, CST has been successfully adapted in many non-English speaking countries including Japan, China, Hong Kong, Italy, Denmark, Nigeria, India, Portugal and Tanzania (International Cognitive Stimulation Therapy Centre, n.d.; Toh et al., 2016). This paper reports the outcomes of a project aimed at culturally adapting CST for Māori. We followed the international guidelines using the formative method for adapting CST to other cultures (Aguirre et al., 2011).

## Materials and Methods

### Setting and Cultural Context

In 2018 Māori stakeholders at Alzheimers Eastern Bay of Plenty approached a New Zealand based CST international trainer about the possibility of adapting CST for Māori in their region. It was considered that the original U.K. manual needed to be reframed to reflect a Māori world view. In addition, wellbeing for Māori is often determined by the balance between their *tinana* (physical), *hinengaro* (mental), *whānau* (family) and *wairua* (spiritual) health (Rochford, 2004); and all major Māori health models emphasise an holistic approach.

Our research team initially comprised a Māori neuropsychologist, a non-Māori old age psychiatrist/international CST trainer and a non-Māori gerontologist/international CST trainer. A Māori clinical psychologist later joined Phase 5 of the adaptation. Under the guidance of elders and key Māori advisors from the local Ngāti Awa tribe in the Bay of Plenty region, the research team invited community stakeholders representative of local tribal affiliations to provide knowledge of relevant historical, cultural and religious/spiritual issues which guided the adaptation of CST for Māori. The adaptation consisted of five phases of the international guidelines (Aguirre et al., 2011): (1) Generating knowledge and collaborating with stakeholders in the Māori community; (2) Integrating generated information with theory and empirical and clinical knowledge; (3) Reviewing the culturally adapted CST intervention by stakeholders, and further revision; (4) Testing the culturally adapted intervention in a pilot study; and (5) Synthesizing stakeholder feedback from the pilot study and finalizing the culturally adapted intervention.

### Phase 1–3: Consultation Meetings with Māori Stakeholders

In 2019 three 1-day *hui* (meetings) over a period of three months were held in Whakatane (a provincial town in the Bay of Plenty). All meetings were conducted according to Māori *tikanga* (protocols) such as beginning and ending with *karakia* (prayer, chants or proverbs), *whakawhanaungatanga* (spending time culturally identifying oneself and making connections with each other), *waiata* (singing traditional songs) and *hākari* (partaking of food).

Over the course of the three meetings, the 18 CST principles and 14 activity sessions were introduced, discussed, and adapted to reflect a Māori world view. In the first meeting with Māori elders it was deemed critical for the CST principles to be grounded in a Māori cultural belief system. In the two subsequent meetings further revision and refinement of the CST principles was undertaken. CST sessions were adapted to include content that were representative and respectful of cultural interests to Māori.

### Phase 4: Pilot Testing the CST-Maōri Programme

Māori with dementia were recruited from Alzheimers Eastern Bay of Plenty to participate in two CST-Māori programmes. Transport was provided for participants to attend the programme. Ethics approval was obtained from New Zealand’s Health and Disability Ethics Committees (Reference 19/NTB/114).

Inclusion criteria were people who: (i) were diagnosed with mild to moderate dementia, defined by a score ≥ 10 on the RUDAS (Rowland Universal Dementia Assessment Scale): (ii) were able to have a “meaningful” conversation; (iii) could hear well enough to participate in small group discussion; and (iv) had vision good enough to see most pictures. People were excluded if they had a recent significant illness, severe visual or hearing impairment, or a terminal illness.

An independent research assistant collected socio-demographic information including the use of medications. The RUDAS and the World Health Organization Quality of Life (WHOQOL-BREF) questionnaire were administered pre- and post-intervention. RUDAS is a brief bedside cognitive screening tool that could be easily interpreted into other languages and be fair across diverse cultural backgrounds (Storey et al., 2004). The WHOQOL-BREF has been shown to be valid for general use in New Zealand (Krageloh et al., 2013).

Due to the small sample size, Wilcoxon Signed Rank Test, a non-parametric statistic test, was used to determine whether there was any statistically significant difference between the pre- and post-outcome measures, with the statistically significant level set at 5%.

Written feedback was sought from *whanau* (extended family) at the end of the CST-Māori programme. We inquired about both the programme’s best and worst aspects, as well as elicited suggestions for improving the programme.

### Phase 5: Consultation with Other Māori Communities and Finalising the CST-Māori Manual

The fifth phase involved five, 1-day *hui* (meetings) with local dementia organisations and Māori social and health services in five regions of the North Island of New Zealand. These regions were selected because of their high numbers of older Māori representing 51% of all older Māori living in New Zealand (Statistics New Zealand, 2020). The purpose of these five meetings was twofold. Firstly, to present the findings from Phase 1–4 of the adaptation; secondly, to sanction the implementation of CST-Māori programme in New Zealand.

## Results

### Phase 1–3

Seven Māori and two non-Māori stakeholders accepted the invitation to support the research team in adapting CST for Maōri. Attendance was varied with four stakeholders committed to all three *hui* (meetings). We provided written feedback about the ongoing consultation and adaptation process to stakeholders who were unable to attend, for example, due to other work commitments. The professional backgrounds of the seven Māori stakeholders and the organisations they represented included a retired general manager of Māori health services in a district health board, a registered nurse in a medical centre, a community health worker and a community nurse in an *Iwi* (tribal) social service, a registered nurse in a district health board memory clinic, a manager and a dementia navigator in a dementia organisation. The two non-Māori stakeholders were a clinical manager who implemented a CST programme in a local dementia day programme and a nurse from a district health board old age psychiatry service.

The research team and the stakeholders jointly decided that an additional session to engage *whānau* (extended family) is necessary at the start of the CST-Māori programme because *whānau* relationships are central to the health and wellbeing of Māori. This additional session also provides an overview of the CST-Māori programme, expected outcomes for the person with dementia, as well as an opportunity for *whānau* to engage in *whakawhanaungatanga* (building connections). We also brought forward the Orientation and Childhood sessions to support further sharing and discussion of participants *whakapapa* (genealogy) in the Orientation session. Table 1 shows the 15 CST-Māori sessions.

To maintain the integrity of the CST programme, we left the structure of each of the 15 CST sessions largely unchanged from the original U.K. manual other than changing the U.K. session content to content that is relevant to Māori culture and New Zealand. For example, in the Orientation session, a map of *Te Ika a Maui* (the North Island of New Zealand) and *Te Waka o Maui* (the South Island of New Zealand) are used. In the Physical Games session, participants play Māori games such as *poi* (a performing art where small light balls on the ends of tethers are swung in rhythmical patterns) and *mau rākau* (skilled use of traditional Māori weapons). In addition, each session follows the Māori meeting protocols that includes a *karakia* (prayer) and *waiata* (singing song) at the beginning and at the end of each session.

The 18 culturally adapted CST principles (Table 2) were presented at the third *hui* (meeting). Since all states of Māori health have a spiritual component, *wairuatanga* (spirituality) underpins all 18 principles of CST and encompasses all that is required to keep the person’s *mana* (prestige) and wellbeing intact, to support them and to provide them with quality experiences.

An initial iteration of the CST-Māori manual was developed based on the culturally adapted sessions and principles.

### Phase 4

The first CST-Māori pilot programme was completed in 2019. However, with the arrival of the COVID-19 pandemic in 2020, the second group was delayed until mid-2021. Two Māori facilitators, who are affiliated to local *iwi* (tribe) and have proficient skills in both Māori and English languages, led both programmes.

A total of 15 Māori (female: 53.3%) participated in the two CST programmes. Their mean age was 75.9 years (SD = 6.6), and their mean baseline RUDAS scores was 17.7 (SD = 2.3). Other information on the participants is presented in Table 3. Table 4 shows the pre- and post-RUDAS and WHOQOL subscale scores. There was a statistically significant improvement in cognition (RUDAS: pre = 17.7, post = 19.4, *p* = 0.003) and in the WHOQOL subscales of physical (pre = 75.9, post = 88.5, *p* = 0.003), psychological (pre = 72.7, post = 81.3, *p* = 0.024) and environment (pre-80.6, post = 88.0, *p* = 0.006).

**Table 3:** Participant socio-demographic and anti-dementia medication information

	*N* = 15
Mean age (SD, range)	75.9 (6.6, 63–87)
Mean RUDAS (SD, range)	17.7 (2.3, 13–21)
Gender: Female	8 (53.3%)
Ethnicity: Māori	15 (100%)
Highest Education	
Less than School Certificate Secondary Tertiary	2 (13.3%) 11 (73.3%) 2 (13.3%)
Lives alone	2 (13.3%)
Marital status	
Married or de facto Widowed Never married	10 (66.7%) 3 (20.0%) 2 (13.3%)
Cholesterase inhibitors ^*^	6 (40.0%)

RUDAS: Rowland Universal Dementia Assessment Scale

*Missing data *n* = 1

**Table 4:** Pre- and post-outcome measures of the 15 participants in 2 Māori cognitive stimulation therapy (CST) programmes

	Pre-CST *N* = 15	Post-CST *N* = 15	*p*-value (Wilcoxon Signed Rank Test)
RUDAS, mean (SD)	17.7 (2.3)	19.4 (2.6)	0.003
WHOQOL-Physical, mean (SD)	75.9 (17.1)	88.5 (8.5)	0.003
WHOQOL-Psychological, mean (SD)	72.7 (14.8)	81.3 (8.8)	0.024
WHOQOL-Social, mean (SD)	69.2 (15.6)	69.6 (13.3)	0.789
WHOQOL-Environment, mean (SD)	80.6 (13.3)	88.0 (9.6)	0.006

RUDAS: Rowland Universal Dementia Assessment Scale

WHOQOL-BREF: World Health Organization Quality of Life

We received 13 written feedback from *whānau* (extended family). Most feedback described how participants enjoyed being in a supportive group environment and participating in cognitive stimulating activities. *Whānau* used the following words to describe the group effect of CST-Māori: W*hakawhanaungatanga* (making connection), company, friendship, and comradeship. A *whānau* reported; “…she was with like-minded people going through the same experiences.” *Whānau* also reported the person with dementia was benefiting from various cognitive stimulating activities during the programme, including learning basics in *Te Reo* (Māori language). One *whānau* reported “…he tried to remember who had attended”; while another *whānau* wrote in the feedback “…she was able to recall a lot of the story where normally she can’t recall much”. Some *whānau* observed an improvement in mood; “…mum always came home cheerful”. In terms of the worst aspect of the programme, most *whānau* were concerned about the time-limiting feature (15 sessions) of CST-Māori. In terms of improving the programme, one *whānau* suggested CST-Māori should be offered at all *marae* (traditional meeting house and surrounds) in New Zealand.

### Phase 5

Five 1-day *hui* (meetings) with local dementia organisations and Māori social and health services were held in 2021. There was general support from participants to take CST-Maōri forward in New Zealand. Since there are many *iwi* (tribes) in New Zealand and it was important to acknowledge *mana whenua* (local tribal authority and traditions). It was agreed that each *iwi* or *hapū* (subtribe) may like to further examine the 18 CST principles and content of CST sessions prior to implementing CST in their region. It was also highlighted that ideally at least one of two CST-Māori facilitators should be fluent in *Te Reo* (Māori language) because some Māori elders speak *Te Reo* as their first language. The cultural identity of a language was also an important factor. A bilingual (English-Te Reo Māori) CST-Māori manual was produced at the end of Phase 5, along with an official launch of the manual on a *marae* (Māori meeting house and surrounds) in 2023.

## Discussion

This study adapted CST for Māori by following internationally recognised guidelines. The feasibility and adaptability of CST has been investigated in other non-English speaking countries such as Tanzania, Nigeria, India and Portugal (Alvares-Pereira et al., 2021; Mkenda et al., 2018; Raghuraman et al., 2017; Wong et al., 2018). However, this is one of the first studies adapting CST to an indigenous population in a bicultural country. In New Zealand CST-Māori is the first manualised psychosocial intervention available for Maōri living with dementia. Our pilot study (Phase 4) found preliminary evidence for CST in improving cognition and aspects of quality of life in Maōri with mild to moderate dementia, although these results are subject to the potential bias of lacking a comparison group.

Previous CST adaptation in other countries or cultures typically focused on linguistic issues and to ensure the 14 CST sessions are culturally appropriate (Alvares-Pereira et al., 2021; Mkenda et al., 2018; Raghuraman et al., 2017; Wong et al., 2018). Our adaptation of CST to Māori included a critical examination of the 18 CST principles through a cultural lens and the Māori world view. We believe Māori cultural belief systems and values are required to be accurately reflected in these principles. As part of adapting CST to Māori, the 18 principles were reviewed by cultural advisors and modified according to a Māori world view which better reflects Māori belief systems and values. For example, person-centred was better understood as *Mana Tāngata* (the uniqueness and status of people), *Whakaute* (respect), *Mahi Ngātahi* (involvement), *Whakakotahitanga* (inclusion), *Tino Rangatiratanga* (maximising potential) and *Whakawhanaungatanga* (building/strengthening relationships). We have also modified the structure of CST to include an initial additional session to engage participants with dementia and their *whānau* (extended family) culturally through *whakawhanaungatanga* (mutual sharing of connections and genealogies), to provide them with an overview of CST-Māori, and expected outcomes for the person with dementia.

The two CST-Māori pilot programmes were well received by *whānau* (extended family) of Māori with dementia, a finding that is similar to CST adaptation in other countries or cultures. (Alvares-Pereira et al., 2021; Mkenda et al., 2018; Raghuraman et al., 2017; Wong et al., 2018). Whānau recognised the positive effects for the person with dementia through engagement in cognitive stimulating activities in a group setting. Feedback from our *whānau* is consistent with the findings of previous qualitative CST studies, which included being with others, practical activities and key outcomes of cognition, enjoyment and mood (Gibbor et al., 2021). Previous studies have identified the importance of carer engagement by providing transport for participants to attend a CST programme and psychoeducation for carers to learn more about the CST sessions (Bertrand et al., 2018; Spector et al., 2011). Our pilot study in Phase 4 successfully engaged *whānau* by including them in the first CST session and providing the participants with transport. Connection and reciprocity are an important value in Māori culture. The group format of CST promotes connection and therefore aligns with traditional Māori values around whanaungatanga. There was also a sense of optimism in the feedback provide by our *whānau*. Dudley et al. (2019) found *whānau* are crucial for the care of a Māori elder with dementia, along with promoting healthy *wairua* (spirit) for all. The *whānau*-inclusive nature of CST-Māori has the potential to improve the wellbeing of both the person with dementia and *whānau*. Future CST-Māori trial could include measuring carer stress or burden as an outcome.

The main strengths of this CST adaptation study lie in engaging with multiple Māori stakeholders over an extended period and adhering to internationally recognised guidelines that have been used successfully to adapt CST in other countries and cultures. We partnered with Māori stakeholders at all five phases of the adaptation and followed Māori *tikanga* (protocols). We also had Māori belief systems and values at the core of the adaptation. Our adaptation was endorsed by the Māori community which will enhance the future adoption of CST-Māori as a post-diagnostic intervention for dementia in New Zealand. Indigenous populations from other parts of the world could use their own cultural protocols, belief systems and values to adapt CST. The main limitation is that the effectiveness of this newly adapted CST-Māori will need to be tested in an adequately powered clinical trial. We consulted five *iwi* (tribes) in Phase 5; we could include other *iwi*, particularly in the lower half of the North Island and the South Island of New Zealand, for further consultation.

## Conclusion

Until we find a cure for dementia and more effective pharmacological treatment, research efforts should continue to focus on improving the quality of life, wellbeing and life satisfaction of people currently living with dementia. Despite the increasing presentations of dementia in Māori, there is a lack of culturally appropriate care following diagnosis. CST-Māori will benefit Māori with dementia. There is also an urgent need for collaborative healthcare practice and practitioners to understand the management and treatment of dementia by accessing the necessary *mātauranga Māori* (Māori knowledge) to provide culturally appropriate and comprehensive care for *whānau*.

## Data Availability

The data for this study is not publicly available to protect the confidentiality of the participants.
